# Les naevus congénitaux géants: à propos d'un cas

**DOI:** 10.11604/pamj.2013.15.156.2978

**Published:** 2013-08-30

**Authors:** Fatima Zahra Hajji Ouafi, Leila Benzekri

**Affiliations:** 1 Service de Dermatologie, CHU Ibn Sina, Rabat, Maroc

**Keywords:** Naevus congénital, plaque pigmentée, mélanome, congenital nevi, pigmented plate, melanome

## Image en médecine

Nous illustrons à travers cette observation le cas d'un nouveau-né qui présente un nævus congénital géant. Il s'agit d'un nouveau-né de 3 semaines de vie de sexe masculin, issu d'un mariage non consanguin, et d'une 1ère grossesse menée à terme, qui présente depuis sa naissance une plaque pigmentée asymptomatique d'une taille entre 12cm/10cm siégeant au niveau de la face antérieure du tronc parsemée de quelques espaces sains, associée à des lésions similaires au niveau de la face dorsale de la main droite et 2 autres lésions au niveau du dos en regard de la colonne vertébrale. Le diagnostic retenu était celui d'un nævus congénital géant. Les nævus congénitaux de grande taille sont exceptionnels 1/2000 à 10000 naissances. La hantise est la transformation maligne en mélanome. D'ailleurs le mélanome est dans 30% des cas associée à un nævus congénital géant et est le plus souvent précoce (10 premières années de vie) et son pronostic est généralement catastrophique. La 2ème complication est la mélanose neuroméningée qui est une manifestation rare qui associe des manifestations pigmentaires à la fois cutanés et méningoencéphaliques. L'atteinte du système nerveux se manifeste par une hydrocéphalie et par des signes de localisation indiquant la réalisation d'une IRM. Le traitement des nævus congénitaux est très difficile. Les excisions itératives sont proposées associées à des techniques d'expansion cutanées. Les alternatives sont la dermabrasion, le laser CO2 et le curetage en période néonatale. Les nævus congénitaux géants sont exceptionnels, de pronostic fâcheux et sont de prise en charge thérapeutique difficile.

**Figure 1:**
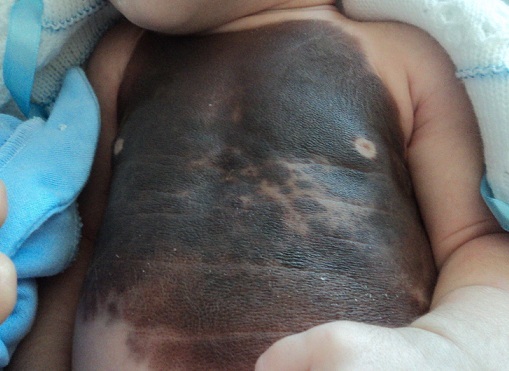
Nævus congénital géant du tronc

